# Remdesivir administration for SARS-CoV-2 pneumonia in ICU and non-ICU patients: outcome and posttreatment differences — the Italian Military Hospital experience

**DOI:** 10.1186/s44158-023-00114-6

**Published:** 2023-09-15

**Authors:** Antonio Sabba, Giancarlo Pontoni, Maria Santangelo, Nadir Rachedi, Maurizio D’Ercole, Bernardino Marseglia, Marcella Fusaro, Elena Giovanna Bignami, Costantino Fontana

**Affiliations:** 1 Rome Military Hospital-Policlinico Militare Di Roma, Rome, Italy; 2 Foligno Army Selection Center, Foligno, Italy; 3 University of Parma, Parma, Italy

**Keywords:** Covid-19, Remdesivir, Intensive care

## Abstract

**Background:**

Four-hundred forty-nine patients affected by Covid-19 were hospitalized at the Rome Military Hospital between March 2020 and July 2022. Depending on the severity of the disease, they were assigned either to the Functional Health Emergency Unit — if suffering from interstitial pneumonia with a clinical manifestation of dyspnea associated with peripheral oxygen saturation  < 92%, and oxygen atmospheric pressure therapy — or to the intensive care unit — if the blood gas-lytic index P/F (ratio between partial pressure of arterial O2 and inspired fraction of O2) was below 150. This prospective observation and monocentric study aim to verify the outcome (healing/death) of early use of remdesivir in pneumonia patients.

**Results:**

The results highlight the importance of the adoption of remdesivir in the initial stages of infection to prevent the systemic spread and viral multiplication and, in the subsequent phase, a cytokine storm resulting in acute respiratory failure and multiorgan failure. The use of the drug in the most advanced stages of the disease is not associated with a real impact on patient outcomes. Therefore, there is a statistically significant correspondence between the early use of remdesivir in the treatment of SARS-CoV-2 disease — in addition to guidelines therapies — and a favorable clinical outcome.

**Conclusions:**

This work shows therapeutic efficacy in the first 5 days of intravenous administration of remdesivir, following the loading dose. It is also necessary to underline the different behaviors of the drug when administered late in patients undergoing mechanical ventilation, compared to those who only needed low-flow oxygen therapy, whose share of recovery — decidedly relevant — reaches statistical significance.

## Introduction

On 11 March 2020, the World Health Organization (WHO) characterized the international outbreak of new virus (SARS-CoV-2) and related the infection as a pandemic [1].

Airborne transmission from an active case is the most common spread mechanism. Transmission between asymptomatic patients is possible but less common [2].

The incubation period can last up to 2 weeks. In the early phase of infection, patients usually present mild and common flu-like symptoms [3]. The radiological findings are similar to other viral pneumonia [4].

The early stage of SARS-CoV-2 infection present flu-like symptoms in 90% of cases, and it is characterized by fever (> 90% of pts and could reach 39 °C or more), cough (dry cough in 45–80% of pts, productive cough in 28%), sickness (44–80% of pts), dyspnea (20–50% of pts, half of them developed dyspnea within 8 days), throat ache (5% of pts), headache (3–20% of pts), and myalgia (11–23% of pts) [5].

During Covid-19 progression, 15–30% of patients developed acute respiratory distress syndrome (ARDS, median 9 days) and needed intensive care unit (ICU) admission and respiratory support treatments [6].

Illness progression was more common in patients with baseline-associated disease, especially in patients who presented arterial hypertension and diabetes mellitus [7].

ARDS is a serious medical condition and need to be treated with life support technique as noninvasive and invasive mechanical ventilation and positive pressure ventilation in order to improve pulmonary gas exchange. In extreme conditions and in selective patients, extracorporeal membrane oxygenation (ECMO) should be considered [8].

Remdesivir is an oral prodrug, nucleotide analogue of adenosine. It is metabolized into the host cells to the active triphosphate form, which is the active form of the parent compound. The active form acts as adenosine triphosphate (ATP) competitive substrate. When remdesivir replaces ATP into the new RNA (made by SARS-CoV-2 RNA-polymerase RNA dependent), it causes subsequent chain termination during viral RNA replication [9].

Remdesivir has efficacy against SARS-CoV-2 infection in vitro on primary human respiratory epithelial cells [10].

The aim of the present study is to assess the remdesivir efficacy in reducing mortality when administered in patients undergoing O2 standard and mechanical ventilated treatments.

## Methods

All consecutive patients admitted to “Celio” Military Polyclinic in Rome during the study period with positive SARS-CoV 2 swab were evaluated for enrollment.

The study was authorized by local ethical committee. All recruitable patients were informed about nature of the study and any aspects including remdesivir administration, data collection, and follow-up. A written informed consent was obtained from all subjects.

The inclusion criteria included the following: age  > 18 years old, positive swab for SARS-CoV-2, and S*pO2* < 92% in room air.

The exclusion criteria included the following: allergy to the active substance or to any other ingredient of remdesivir, patients with stages 4 and 5 of renal failure (defined as estimated glomerular filtration rate  < 30 ml/min), patients with markedly increase in transaminases (ASAT and ALAT 5 times above the lab limit), and pregnancy.

Stable patients — defined as patients presenting pneumonia, *SpO2* < 92% in room air and clinical improvement after O2 standard therapy — were admitted to UFES. Unstable patients — defined as patients who presented PaO2/FiO2 < 150 — were admitted to ICU.

All patients received best available treatments including low-molecular-weight heparin 100 UI/kg/day, dexamethasone 6 mg/day for 10 days or methylprednisolone 40 mg/day for 10 days, and acetaminophen 1 g when body temperature raised over 38 °C.

Remdesivir was available from 12–05-2020. All consecutive patients enrolled in this study after 12–05-2020 received remdesivir on top of the best available treatments.

The control group is represented by patients that fit inclusion criteria and were admitted to the hospital before remdesivir approval.

In control arm, patients received best available treatments plus lopinavir/ritonavir (400/100 mg twice a day for 7 days), hydroxychloroquine (200 mg twice a day) when necessary, or tocilizumab (same doses above).

In treatment group, patients received best available treatments plus remdesivir for 5 days (200 mg loading dose the day of enrollment and 100 mg/day for next 4 days). Four cases — characterized by serious clinical condition and rapidly worsening — were treated with remdesivir (same doses above) and tocilizumab (400 mg one shot).

### Statistical analysis

Qualitative variables were expressed as number and percentages. Quantitative variables were reported as mean, median, standard deviation (DS), and range.

Frequencies and contingency tables were used to describe categorical variables.

Baseline data included age, sex, comorbidities, admission ward, and other clinical parameters (transfer to ICU, necessity of tracheal intubation, etc.).

Unadjusted univariate analyses was based on Pearson chi-square test for categorical variables. Continuous variables were analyzed by means *t*-test, in accordance with normality of the distribution.

A logistic regression model was used to evaluate study primary endpoint.

At first, all nonbinary covariates (age and number of comorbidities) were dichotomized according to their median, and univariate logistic regressions were performed. Variable response was patients’ outcome (survive or death). Therefore, two multivariate logistic models were built, one for each group including the covariates that had reached the statistical significance at the univariate analysis.

Odds ratios (OR) were used as a measure of effect size. A *p*-value of 0.05 or lower was considered as statistically significant.

Statistical analysis was performed using the software STATA version 14.2 (StataCorp LLC, College Station, TX, USA).

### Sample size estimation

The sample size estimation was performed using the dedicated software made available by the Cleveland Clinic — Department of Quantitative Health Sciences available at http://riskcalc.org/samplesize/. The minimum number of subjects required for this study was estimated considering a 5% margin of error, a 95% confidence level, a sample size ratio between the two groups of 3, and a study power of 90% (1 beta = 0.9). From the literature available in Italy at the moment of this study design, we estimate a proportion of deaths for ICU and for all ward hospitalized patients of 0.26 and 0.53, respectively.

Based on the above calculation and considering the estimated sample size ratio, the minimum number of observations to be made is 200 divided into 50 belonging to the ICU group and 150 to the FHEU group.

## Results

We enrolled 449 patients affected by Covid-19 admitted to “Celio” Military Polyclinic in Rome between March 2020 and July 2022.

Stable patients, admitted to UFES, were 334 (74%), while unstable patients admitted to ICU were 115 (26%) (Table 1).

**Table 1:** Main demographic, hospitalization, and clinical features at the time of hospitalization

**Demographic and hospitalization features**^**a**^	*n* **(number)**	**% (percentage)**	**Clinical features**^**a**^	*n* **(number)**		**% (percentage)**
*Age class (years)*			*Number of comorbidities*			
24–60	222	49.4	0		143	31.8
61–93	227	51.6	1		87	19.4
			2		56	12.5
*Sex*			3 or more		163	36.3
Male	339	75.5				
Female	110	24.5	*Pharmacotherapy*			
			None		104	23.2
*Ward at admission*			Remdesivir		262	58.4
UFES	334	74.4	Tocilizumab		6	1.3
ICU	115	25.6	Tocilizumab + remdesivir		4	0.9
			Tocilizumab + Kal		3	0.7
*ICU transfer*			Kal + ClK		42	9.4
Yes	23	6.9	Kal		15	3.3
No	311	93.1	ClK		13	2.9
*IOT (ICU patients)*			*Outcome*			
Yes	67	58.3	Survive		354	78.8
No	48	41.7	Death		95	21.2

^a^Numbers may not add up to 449 because of missing data. Percentages may not add up to 100 because of rounding

The average age was 62.0 years old (range 24–29; *DS* 14.7), and males represented most patients admitted (*n* = 339, 75.5%). Two-hundred thirty patients (51%) presented 1 or less comorbidity, while 49% presented two or more comorbidities. Twenty-three stable patients (7% of UFES pts) admitted to UFES required the ICU admission after worsening clinical conditions. Endotracheal intubation was necessary in 67 patients (58% of ICU pts) admitted to ICU.

One-hundred four patients (23%) did not receive any antiviral therapy. In our hospital, remdesivir was the most used antiviral drug (*n* = 266, 59% of patients). Only 4 patients (less than 1% of total pts) received remdesivir plus tocilizumab. Three-hundred fifty-four patients (79%) survived Covid-19 pneumonia.

The UFES and ICU groups showed heterogeneity for age, sex, and number of comorbidities. Mean difference of 8.2 years was found between the UFES and ICU groups, with statistical significance (*p* < 0.001). After the age dichotomy based on the median value of 60 years, 38% (*n* = 89) of patients over 60 years of age versus 13% (*n* = 26) of patients under 60 were admitted to treatment intensive, and this difference reached a level of *p* < 0.01 statistical significance.

Male patients were admitted to intensive care in 64% (*n* = 74) of cases while female patients in 36% (*n* = 41) of cases, and the difference was statistically significant (*p* = 0.001).

Patients with none or at least one comorbidity condition were admitted to intensive care in 19% (*n* = 44), while patients with two or more comorbidities were admitted to intensive care in 32% (*n* = 71) of cases with a statistically significant difference (*p* = 0.001).

Remdesivir was administered in 61% (*n* = 203) of UFES patients and 55% (*n* = 64) of ICU patients, but this difference was not statistically significant (*p* = 0.26). Remdesivir was more prescribed in patients less than 60 years old (65%, *n* = 145) than in those over 60 (53%, *n* = 121) and in patients with none or at least one comorbidity (67%, *n* = 155) versus two or more comorbidities (51%, *n* = 112). These differences reach a statistical significance level of 0.01 and < 0.001, respectively. In intensive care, remdesivir was administered in 43% (*n* = 29) of patients who undergone IOT and in 71% (*n* = 34) of patients in NIV, with a statistically significant *p* = 0.003.

**Fig. 1:**
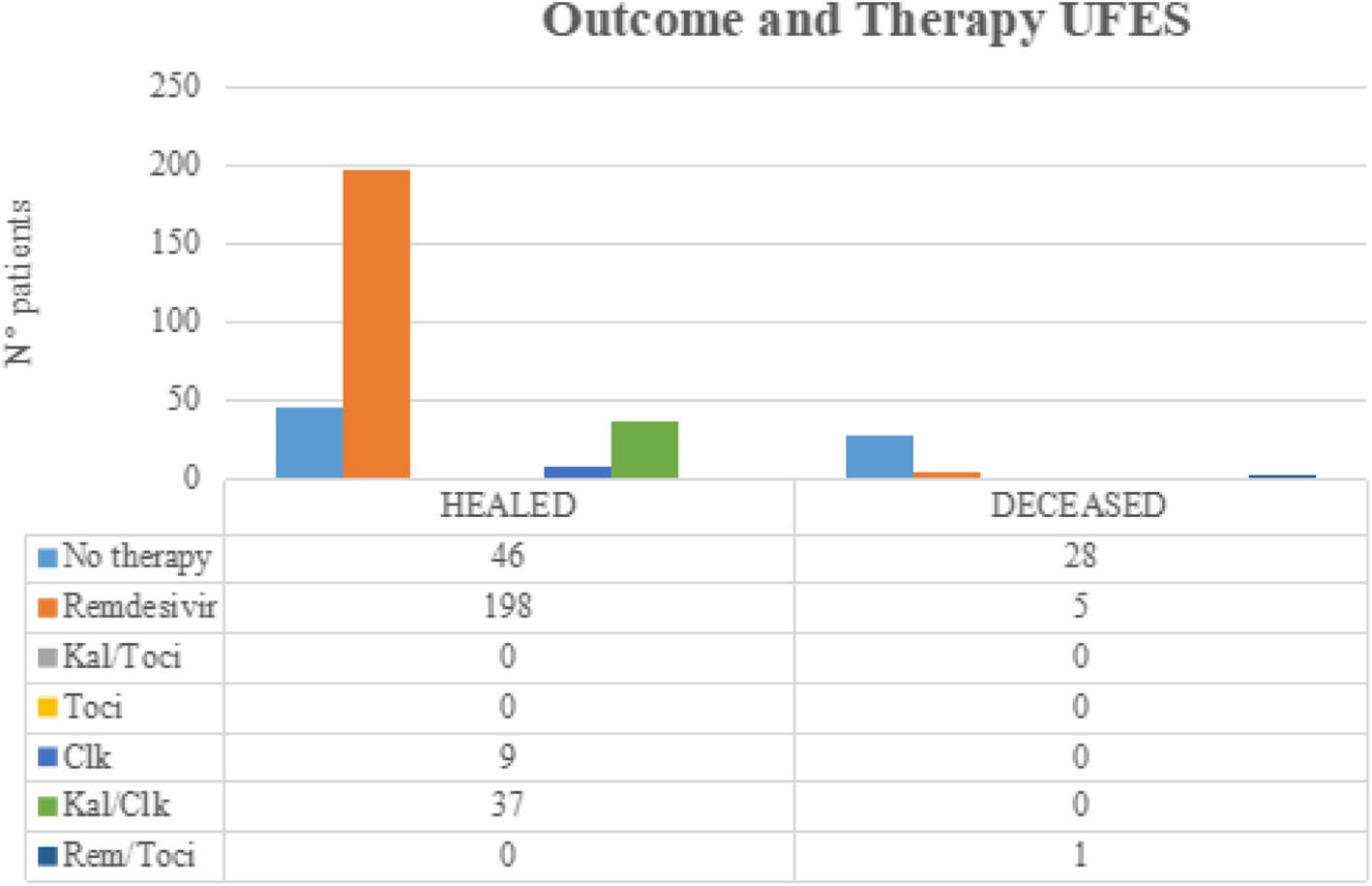
Outcome and therapy UFES

**Fig. 2:**
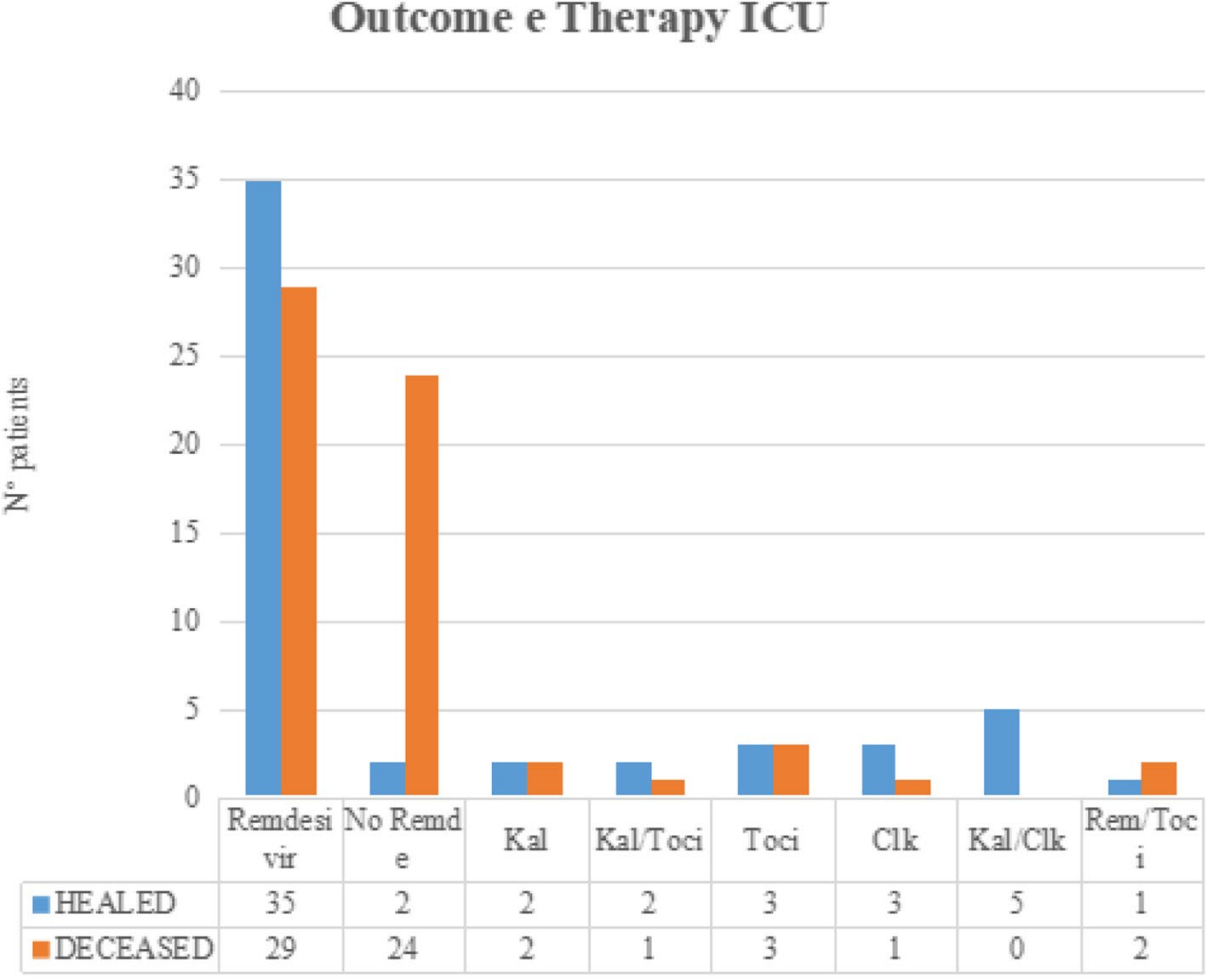
Outcome therapy ICU

There is no significant difference (*p* = 0.25) when comparing remdesivir prescriptions in male (61%, *n* = 206) and female (55%, *n* = 61) patients (Figs. 1 and 2).

A total of 90% of UFES patients survived (*n* = 300), and 53% of ICU patients (*n* = 61) survived. Males survived in the 83% (*n* = 283) of cases while female patients in the 65% (*n* = 71). The 98% of patients less than 60 years old survived (*n* = 217) while patients older than 60 years of age in the 60% (*n* = 137) of cases. A total of 88% of patients treated with remdesivir survived (*n* = 233) while the remaining in 66% (*n* = 121) of cases. A total of 91% of patients with none or at least one comorbidity condition survived (*n* = 210), while patients with two or more comorbidities survived in 66% (*n* = 144) of cases. Each of the last five differences reached a level of statistical significance (*p* < 0.001). Univariate logistic regression analysis was performed for each group. Univariate analysis of the UFES group showed a negative association between remdesivir treatment and death (*OR* 0.09, *p* < 0.001) and a positive association of age  > 60 (*OR* 58, *p* < 0.001), female sex (*OR* 3, *p* = 0.03), two or more comorbidities (*OR* 53, *p* < 0.001), and death (Table 2). Univariate analysis of the ICU group showed a negative association between remdesivir treatment and death (*OR* 0.5, *p* = 0.04) and a positive association between age  > 60 and death (*OR* 12, *p* < 0.001). Multivariate logistic regression analysis of the UFES group showed a negative association with remdesivir and death (*OR* 0.1, *p* < 0.001) and a positive association of age  > 60 (*OR* 20, *p* = 0.005), two or plus comorbidities (*OR* 12, *p* = 0.01), and death. Multivariate analysis of the ICU group showed a loss of significance of all co-varied, except for  > 60 years of age. Older age remains the only independent predictor of death (*OR* 11.6, *p* < 0.01) in this group.

**Table 2:** Multivariate logistic regression analysis of factors associated with death in UFES patients

**Death**	**OR (95% ***CI***)**	*p*
Treatment with remdesivir	0.10 (0.04–0.30)	< 0.001*
Comorbidities (2 or more)	13.58 (1.68–109.95)	0.014*
Sex (male)	1.89 (0.73–4.92)	0.190
Age (> 60 years)	20.4 (2.53–164.41)	< 0.005*

^*^Statistical significance (*p* < 0.05)

## Discussions

The results of this study highlight the importance of remdesivir in the early stages of infection, to prevent systemic viral spread and multiplication from triggering a cytokine storm, which can lead to a fatal acute respiratory and multiorgan failure [9]. It is clear that the early administration of remdesivir in patients in the early stages of infection (within 10 days from the first positive) can generate various clinical benefits, reducing the hospitalization as well as the consumption of health resources because it reduces the access to intensive care units. The statistical analysis of the available data confirmed the efficacy of remdesivir in the early stages of Covid-19. However, it demonstrates that the use of the drug in the most advanced stages of the disease is not associated with a real improvement on patients’ outcome. The study design shows that some subgroups of patients — particularly the group under 60 — had clear clinical benefits if remdesivir was administered on the onset phase [11, 12]. The remdesivir active principle is in fact recognized in a highly water-soluble molecule that shows poor pulmonary diffusion which is why the therapeutic window of efficacy appears to be closely related to tissue distribution pharmacokinetics during the phase that immediately precedes the parenchymal damage. This work shows therapeutic efficacy in the first 5 days of intravenous administration of remdesivir, following the loading dose. It is also necessary to underline the different behaviors of the drug when administered late in patients undergoing mechanical ventilation, compared to those who only needed low-flow oxygen therapy, whose share of recovery — decidedly relevant — reaches statistical significance [8, 13]. We have to underline that the control group belongs exclusively to the so-called first pandemic wave, while the patients of the treatment group were enrolled until July 2022. In our opinion, this is not a limitation for this observational study for several reasons. In particular, it is important to underline that we do look at the Covid-19 pandemic a unique event as described by the WHO, and we do consider the waves concept to much linked with geographically sensitivity, and we did not change our approach. Moreover, we did not enroll patients vaccinated against Covid-19, and even considering the different timing of enrollment between the control and treatment groups, it might be useful to underline that the best available treatment algorithm has not changed over time [14, 15].

The study shows that the timely use of remdesivir in addition to the other standard therapies — considering the results conducted in a single center — leads to favorable clinical outcomes guaranteeing a statistically significant cure and avoids the unfortunate evolution of the disease, particularly in patients not undergoing mechanical ventilation and under the age of 60. However, a certain clinical efficacy — though not significant — should be remarked even in patients under mechanical ventilation [16]. Conclusions are similar to literature constituted, and this is a further confirmation of the clinical efficacy of remdesivir in the treatment of SARS-CoV-2 disease. We can conclude that the early administration of remdesivir in patients in the early stages of infection (within 10 days from the first positive) can generate various clinical benefits. This observational study emphasized that the benefit of remdesivir appears to be linked to the precociousness of the treatment and the timing of administration with respect to the admission and from the first positivity.

### Take-home message

The early administration of remdesivir in patients in the early stages of infection (within 10 days from the first positive) can generate various clinical benefits. This observational study emphasized that the benefit of remdesivir appears to be linked to the precociousness of the treatment and the timing of administration with respect to the admission and from the first positivity.

### Tweet

The early use of remdesivir in the treatment of SARS-CoV-2 disease — in addition to guidelines therapies — generates a favorable clinical outcome.

## Data Availability

We can provide all the data we collected for this study.
